# Effects of a targeted resistance intervention compared to a sham intervention on gluteal muscle hypertrophy, fatty infiltration and strength in people with hip osteoarthritis: analysis of secondary outcomes from a randomised clinical trial

**DOI:** 10.1186/s12891-022-05907-4

**Published:** 2022-10-29

**Authors:** Zachary P. J. Rostron, Anita Zacharias, Adam I. Semciw, Michael Kingsley, Tania Pizzari, Stephanie J. Woodley, Rodney Green

**Affiliations:** 1 Department of Rural Clinical Sciences, La Trobe Rural Health School, La Trobe University, Edwards Rd, Flora Hill, PO Box 199, Bendigo, VIC 3552 Australia; 2 Department of Physiotherapy, Podiatry, and Prosthetics and Orthotics, School of Allied Health La Trobe University, Bundoora, VIC Australia; 3 School of Health and Rehabilitation Sciences, The University of Queensland, St Lucia, QLD Australia; 4 Department of Exercise Sciences, Faculty of Science, University of Auckland, Auckland, New Zealand; 5 Holsworth Research Initiative, College of Science, Health and Engineering, La Trobe University, Bendigo, VIC Australia; 6 Department of Anatomy, School of Biomedical Sciences, University of Otago, Dunedin, New Zealand

**Keywords:** MRI, Muscle volume, Muscle strength, Degenerative Arthritis, Rehabilitation, Exercises

## Abstract

**Background:**

People with hip osteoarthritis are typically offered a combination of education and exercise to address muscle atrophy and weakness. Limited evidence exists to assess the efficacy of exercise programs on muscle structure or function in this population. The aim of this study was to evaluate the effects of targeted resistance exercise on gluteal muscle hypertrophy and strength in people with mild-to-moderate hip osteoarthritis.

**Methods:**

Twenty-seven participants with radiologically confirmed hip osteoarthritis recruited from a single site of a multi-site, double-blind clinical trial were randomly allocated to receive a 12-week targeted gluteal intervention or sham intervention. Magnetic resonance imaging and hand-held dynamometry were used to determine change in gluteal muscle volume, fatty infiltration and hip muscle strength. For gluteal muscle volume and strength outcomes mixed model analyses of variance (ANOVA) were conducted. A general linear model (ANOVA) analysis with fixed effects parameter estimates was used to assess the impact of sex on gluteal muscle size and strength of the affected limb only. For muscle fat index a mixed method ANOVA was used to assess the differences between groups and over time.

**Results:**

In the targeted intervention group, gluteus minimus volume increased from baseline to post-intervention in both limbs (pooled mean difference: 0.06 cm^3^/kg, 95% confidence interval: 0.01 to 0.11) while no change occurred in the sham group (time x group effect: *P* = 0.025). Gluteus medius, gluteus maximus and tensor fascia lata volume did not change significantly over time. Hip strength (abduction, adduction, flexion, extension, external and internal rotation) improved similarly in both groups (time main effect: P ≤ 0.042). There was a consistent, albeit non-significant, pattern of reduced fatty infiltration after the targeted intervention.

**Conclusion:**

Targeted resistance exercise resulted in gluteus minimus hypertrophy, but improvements in hip strength occurred in both groups. Clinicians delivering hip osteoarthritis rehabilitation programs might consider implementing a targeted exercise program to attenuate disease associated changes within gluteal muscles.

**Trial registration:**

Australian New Zealand Clinical Trials Registry, ID: ACTRN12617000970347. Registered prospectively on 5 July 2017.

**Supplementary Information:**

The online version contains supplementary material available at 10.1186/s12891-022-05907-4.

## Introduction

Evidence-based clinical guidelines for the conservative management of people with hip osteoarthritis (OA) recommend education and physical activity or exercise prescription [1]. People with hip OA exhibit reduced physical activity, which might contribute to gluteal muscle atrophy, increased fatty infiltrate and weakness identified in symptomatic hips [2]. High-intensity resistance exercise programs improve hip abduction strength in people with hip OA, but it is unclear if this improvement in muscle strength is associated with muscle hypertrophy [3].

At the hip joint, gluteus minimus (GMin), medius (GMed) and maximus (GMax) act as prime movers and stabilisers. In recent years there has been a focus on changes in gluteal muscles that are associated with hip OA [2, 4–7], and the most suitable exercise program to attenuate muscle deterioration [8, 9]. Muscle atrophy and high levels of fatty infiltration are present in the gluteal muscles of people with hip OA, particularly in the GMin, with higher levels of fatty infiltration observed in those with more severe disease [10]. The degree of fatty infiltration is important because concomitant loss of contractile muscle results in a lowered capability to produce force (i.e., reduced muscle strength) [11].

Resistance based exercise interventions are associated with muscle hypertrophy in the general population [12], and there is evidence of exercise-induced hypertrophy in quadriceps [13] and gluteal muscles [14] of people with hip OA. Greater decreases in intramuscular fat are observed following a high-velocity exercise intervention when compared to a low-velocity intervention [14]. A targeted resistance exercise program might address the known deficits in the periarticular muscles of people with hip OA, when GMin is particularly affected.

The aim of this analysis was to investigate whether an exercise program targeting the gluteal muscles is more effective than a sham exercise intervention in producing muscle hypertrophy, reducing fatty infiltration and increasing strength in people with mild-to-moderate hip osteoarthritis. A secondary aim was to evaluate the impact of sex on any changes in gluteal muscle size and strength.

## Methods

### Design

**Fig. 1:**
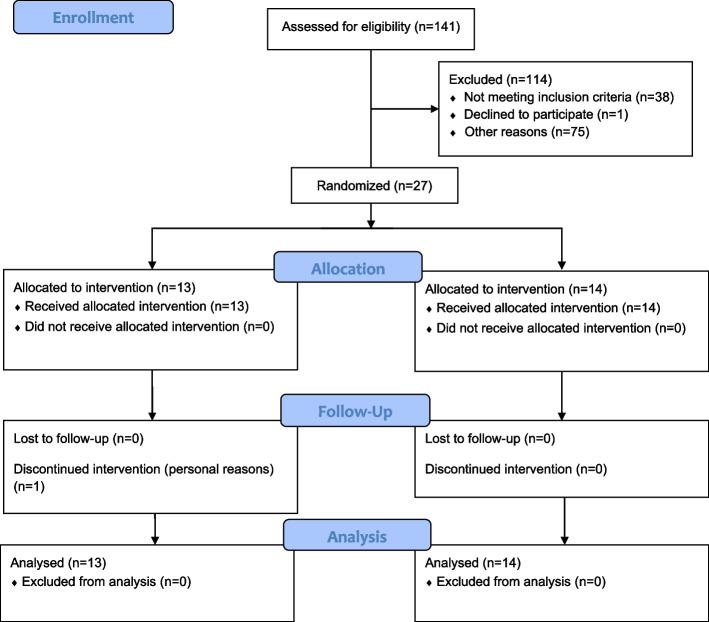
Flow diagram of participants

Participants were recruited to this embedded study from a single site (Bendigo, Australia) of a larger multi-site double-blinded randomised controlled trial (the GHOst trial – Gluteal exercise for Hip Osteoarthritis) [15], registered 05/07/2017 on the Australian New Zealand Clinical Trials Registry (ACTRN12617000970347). This paper reports on secondary outcome measures (i.e., muscle volume, fatty infiltration and hip muscle strength) from the trial. After eligibility was confirmed using a two-stage screening process (phone interview followed by face-to-face physical assessment with a musculoskeletal physiotherapist), baseline measures of magnetic resonance imaging (MRI), hip strength and anthropometry were conducted. Participants were randomly allocated to receive either a 12-week targeted gluteal exercise program or a 12-week sham exercise program (Fig. 1) [15]. Post-intervention, participants underwent a follow-up MRI and hip strength measures.

### Participants

Participants with hip OA (radiologically confirmed unilateral or bilateral hip OA, Grade ≥ 2) [16] were recruited via flyers and online advertising services. After screening for eligibility, participants with mild-to-moderate disability from hip OA were included [15], as indicated by an Oxford Hip Score (OHS) of 25 to 45, which is a reliable score of patient-reported outcome measures of hip-related disability [17]. Participants were excluded if they reported any musculoskeletal or other medical conditions that might be exacerbated by intense exercise or a contraindication to MRI scans [15]. For participants with bilateral hip OA, the affected limb was defined as the most painful hip and the other was designated as the contralateral limb.

### Interventions

Both interventions were conducted by experienced musculoskeletal physiotherapists and included standard education on hip OA and general physical activity, weekly supervised physiotherapy sessions in individual or small group settings and a self-managed home exercise program that consisted of the same exercises prescribed during the supervised physiotherapy sessions [15].

#### Targeted gluteal intervention

The targeted intervention was designed to help address the known deficits in people with mild-to-moderate hip OA and consisted of multiple stages (stages 1–4) in each of three main phases of rehabilitation: (1) gait retraining, involving but not limited to auditory cueing during gait and backwards walking; (2) GMin muscle strengthening, by incorporating functional progressions of split squat and bridge exercises; and (3) pelvic stability and global strengthening, which involved various high-intensity strength exercises aimed to targeting multiple muscle groups, such as isometric hip hitch, double-leg squat and deadlifts (https://doi.org/10.26181/19897624.v1). Participant progression through the exercise stages was determined by multiple factors including the physiotherapist’s judgment, reaching a target level of intensity measured as a perceived exertion of ≥ 5 (strong) to < 7 (very strong) on the Borg scale [18], participant symptoms, and the participant’s ability to complete the exercise.

#### Sham exercise intervention

The sham intervention was a general low-intensity lower limb multistage (stages 1–3) exercise program, aimed at multiple muscle groups and included unloaded (mostly seated) gluteal, quadriceps and calf exercises [15] (https://doi.org/10.26181/19897624.v1).

### Outcome assessment

Researchers were blinded to group (targeted vs sham) during data collection. During analysis of MRI data, researchers were also blinded to timepoint (baseline vs post-intervention) and limb (affected vs contralateral).

#### Muscle volume

**Fig. 2:**
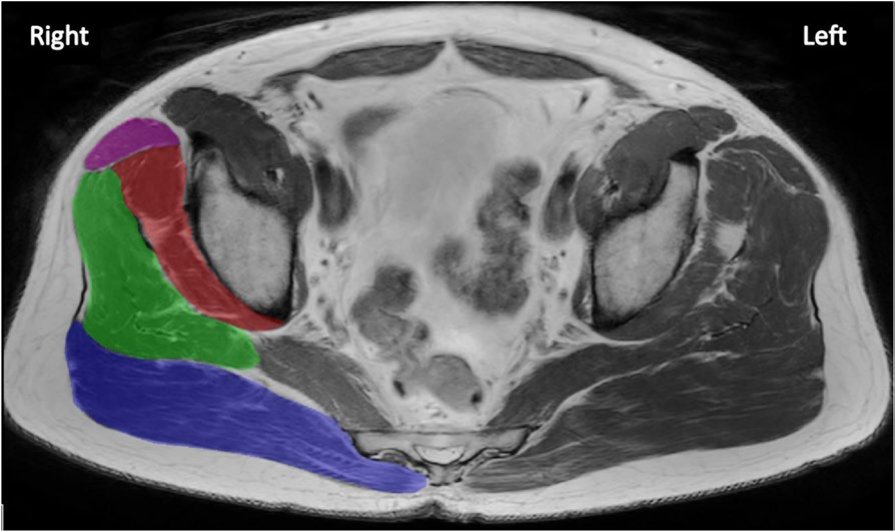
MRI tracings with manually generated masks for muscle volume calculation in an affected right hip. Note. GMax  , GMed  , GMin  , TFL

For MRI, participants were placed supine and their feet were stabilised to avoid hip rotation [2]. A multi-planar localiser scan was conducted from above the iliac crest to the mid femur to specifically identify muscle volume and proportion of fat within the muscles of interest (i.e., GMax, GMed, GMin and TFL) [2]. The MRI (Ingenia 3.0 T; Phillips, Cambridge MA, USA) scan, with all axial images (Ax T1, Turbo Spin Echo (TSE), Ax T2 mDIXON, Coronal (Cor) T1 TSE and Cor T2 mDIXON) was set to consistent parameters (slice thickness: 6 mm × 56 slices, field of view: 260 mm; 308 × 406 matrix, echo time 60 ms, repetition time 3787 ms, total acquisition time: approximately 6 min). The DIXON sequence obtains both fat and water-saturated images and allows for quantification of fat present within a muscle [19, 20]. Individual muscles were manually traced by one of two researchers, following fascial outlines [21] and creating masks to determine the cross-sectional area of the muscle on each slice on both sides (Fig. 2) using ITK-SNAP (Version 3.8.0; University of Pennsylvania, 2018) [22]. Muscle volume was calculated by determining the CSA of each muscle and multiplying it by the slice thickness (6 mm) to define slice volume and then summing across all slices to calculate total muscle volume. Total muscle volume (cm^3^) for GMax, GMed, GMin and TFL was determined for each limb (i.e., affected and contralateral) and was normalised between participants by dividing by baseline body mass (cm^3^/kg)[2]. MRI is a reliable method to measure muscle volume [23]. Inter-rater reliability was determined for this muscle volume measurement for both limbs of five participants using intraclass correlation coefficients (ICC) and interpreted as poor (0.5 ≤ ICC < 0.75), good (0.75 ≤ ICC < 0.90) or excellent (ICC ≥ 0.90) [24]. Excellent ICC values were observed for GMax (0.995), GMed (0.978), GMin (0.940) and TFL (0.980).

#### Muscle fat index

Muscle fat index was calculated to represent the proportion of fatty infiltrate within each muscle for the affected limb. The ‘neurobase’ R package (Version 1.29.0) was used, via R statistical software (Version 4.0.2; RStudio, PBC, Boston MA, 2020), to extract pixel intensity values from the water and fat only images for specific areas of each muscle defined by the previously constructed masks. These values were used to calculate muscle fat index following previously described procedures [25].

Muscle length was normalised [26] to enable analysis of fat over the entire length of the muscle from proximal to distal (0% most proximal slice and 100% most distal slice). Using spline interpolation, the mean muscle fat index was represented at every 1% of muscle length. The GMin and GMed muscles were segmented by division into two (anterior and posterior (GMin) segments) or three (anterior, middle and posterior (GMed) segments) equal portions respectively, due to their structural and functional unique segments [27, 28].

#### Isometric hip muscle strength

Isometric hip strength was measured with a hand held dynamometer [15], using methods previously shown to be reliable [29]. Strength was measured bilaterally for hip abduction, adduction, flexion, extension, internal rotation and external rotation. The test order was randomised for the assessment, and the same sequence was repeated post-intervention. Participants completed two trials (15 s between attempts) for each measure of the contralateral then affected limb. Participants were instructed to slowly push against resistance until they reached their maximum isometric strength and to hold this for three seconds allowing measurement of peak force (N) [2]. Standardised audio recordings were used to encourage maximum effort for all participants and the highest of the two trials was recorded as the maximum strength. The distance between the dynamometer position and the axis of rotation was measured (m) and used to determine moment arms for strength measurements so that they could be expressed as a torque (Nm).

### Statistical analyses

Initial analyses were performed using IBM SPSS Statistics for Windows (Version 27.0; Armonk, NY: IBM Corp,) with the level of statistical significance set at *P* < 0.05. Continuous data were assessed for normality using the Kolmogorov–Smirnov test. If data were not normally distributed, data were then log transformed prior to subsequent analyses. Characteristics of the intervention groups were compared at baseline using independent samples t-tests for parametric data and Chi-square tests for categorical data. Mixed model analyses of variance (ANOVA) were conducted for muscle volume and strength; the between group variable was intervention (targeted or sham) and the within group variables were limb (affected and contralateral) and time (baseline and post-intervention). Interaction effects were consulted to determine if changes in the dependent variable over time differed by intervention or limb. Main effects were consulted when the interaction effect did not reach significance.

To evaluate the secondary aim, the impact of sex on gluteal muscle size and strength (affected limb only), a general linear model (ANOVA) analysis with fixed effects parameter estimates was performed using jamovi (Version 2.2; Sydney AU, 2021; https://www.jamovi.org) with a Bonferroni post hoc analysis completed to evaluate the overall significance of the general linear model. For muscle volume and strength, pooled mean difference (MD) were calculated from baseline to post-intervention (post-intervention – baseline / baseline = pooled MD) with 95% confidence intervals (95% CI) and effect sizes were classified as small (> 0.2), medium (> 0.5) and large (> 0.8) [30] in accordance with Cochrane guidelines [31].

For muscle fat index analysis, where data points exist across every percentile of the length of the muscle, a mixed method ANOVA was completed to examine differences between groups and over time (baseline to post-intervention) [26]. To specifically test for differences in muscle fat proportions over time, and to observe the impact of either intervention, statistical nonparametric mapping (SnMP) was used (open‐source spm1d code [SPM spm1d], Version 0.4; 2021; http://www.spm1d.org]) [6, 26, 32] which results in the calculation of the “t” statistic for each 1% of muscle length allowing the location of differences in muscle fat index over the length of the muscle to be determined. Within the SnMP framework, a nonparametric mixed ANOVA was performed using Python (Version 2.7; 2010, via Canopy, Enthought Inc, version 2.1.9; 2018) [26]. For muscle fat index, effect sizes (ES) were determined by dividing the t‐statistic by the square root of the sample size.

## Results

### Demographic characteristics and comparisons at baseline

Participant characteristics at baseline did not differ between the two groups (Table 1). Of the 27 participants, there were 14 males (targeted: *n* = 7, sham: *n* = 7) and 13 females (targeted: *n* = 6, sham: *n* = 7), aged between 41–75 years. One female in the targeted gluteal intervention did not complete the last five weeks of the exercise program for personal reasons but completed all baseline and post-intervention outcome measures and was included in the analyses. Most participants (> 86%) in the sham group reached the final stage of all phases. All participants in the targeted group completed gait retraining (commenced week 1) and GMin motor control (commenced final stage in week 2), but only 69% reached the final (fourth) stage of the pelvic stability phase (92% reached stage two and stage three). For those participants that reached the final stage of the pelvic stability phase, on average the final stage was commenced in week eight.

**Table 1:** Participant characteristics (mean ± SD unless indicated otherwise) at baseline for the targeted gluteal and sham interventions

	Targeted (*N* = 13)	Sham (*N* = 14)	*P*-value
Age (years)	58.2 ± 10.9	60.1 ± 7.3	0.08^1^
Sex (% female)	46	50	0.84^2^
Height (cm)	173 ± 10	169 ± 10	0.94^1^
Body mass (kg)	88.8 ± 18.2	82.3 ± 15.4	0.29^1^
Body mass index (kg/m^2^)	29.4 ± 4.4	28.9 ± 5.7	0.67^1^
K-L score (Affected limb)			0.86^2^
(% grade 2)	54	57	
(% grade 3)	46	36	
(% grade 4)	0	7	
K-L score (Contralateral limb)			0.74^2^
(% grade 0)	8	14	
(% grade 1)	31	36	
(% grade 2)	46	43	
(% grade 3)	15	7	
(% grade 4)	0	0	
OHS (affected limb)	31.5 ± 4.6	33.4 ± 4.9	0.29^1^

Note. Severity of OA was determined according to the Kellgren and Lawrence, 1957 [16] radiological assessment for OA. Kellgren and Lawrence (K-L) score: 0 (none), 1 (doubtful), 2 (minimal), 3 (moderate) and 4 (severe)

^1^Independent-samples t-test

^2^Chi-square test

### Muscle volume

**Fig. 3:**
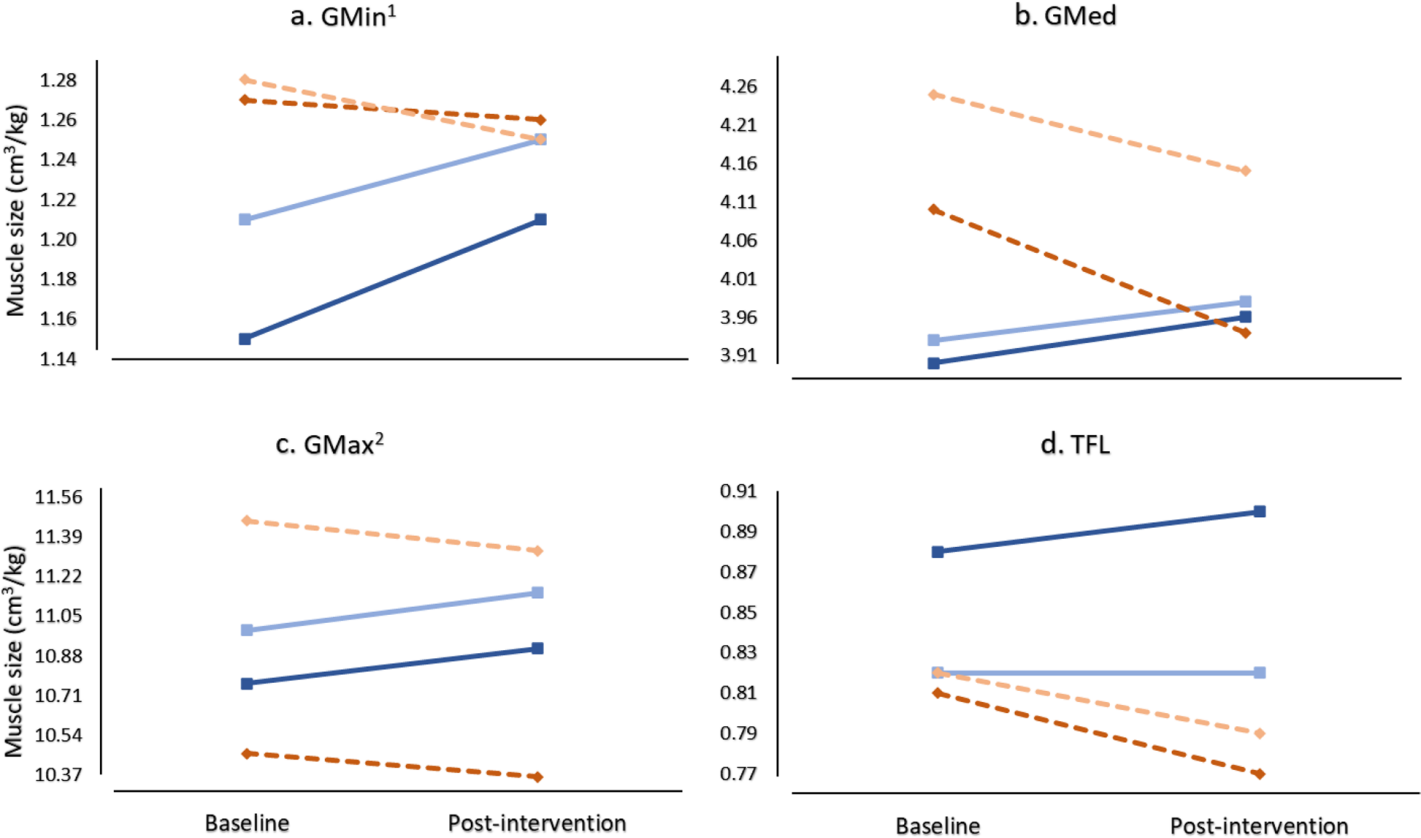
**a**-**d** Mean normalised gluteal muscle size (cm^3^/kg) for the targeted (solid blue lines) and sham interventions (dashed orange lines). Note. Affected and contralateral limbs indicated by darker and lighter lines, respectively (standard deviations in Additional file 1). ^1^Time x group effect (*P* < 0.05); ^2^ Limb main effect (*P* < 0.05). Non-significant trends referred to in the text can be observed by the slope of the lines on each plot

Change in GMin volume from baseline to post-intervention differed by intervention across both limbs (time x group effect: F_1,25_ = 5.70, *P* = 0.025; Fig. 3a), where GMin volume increased following the targeted intervention in both limbs (pooled MD: 0.06 cm^3^/kg, 95% CI: 0.01 to 0.11) with moderate effect sizes (affected ES = 0.70, contralateral ES = 0.87) (see Additional file 1). Consistent, albeit non-significant patterns were observed with either increases for the targeted group and/or decreases for the sham group across both limbs for all other muscles (time x group effect: F_1,25_ ≤ 4.05, *P* ≥ 0.055; Fig. 3b-d) with effect sizes as follows: GMed (affected ES = 0.64, contralateral ES = 0.47), GMax (affected ES = 0.43, contralateral ES = 0.59), TFL (affected ES = 0.94, contralateral ES = 0.40) (see Additional file 1).

Although there were no significant changes over time, GMax muscle volume for the affected limb was smaller compared to the contralateral limb across both time points (limb main effect: F_1,25_ = 15.33, *P* = 0.001, MD: 0.61 cm^3^/kg, 95% CI: 0.23 to 0.93; Fig. 3c). There were no significant differences between limbs for GMin, GMed or TFL.

**Fig. 4:**
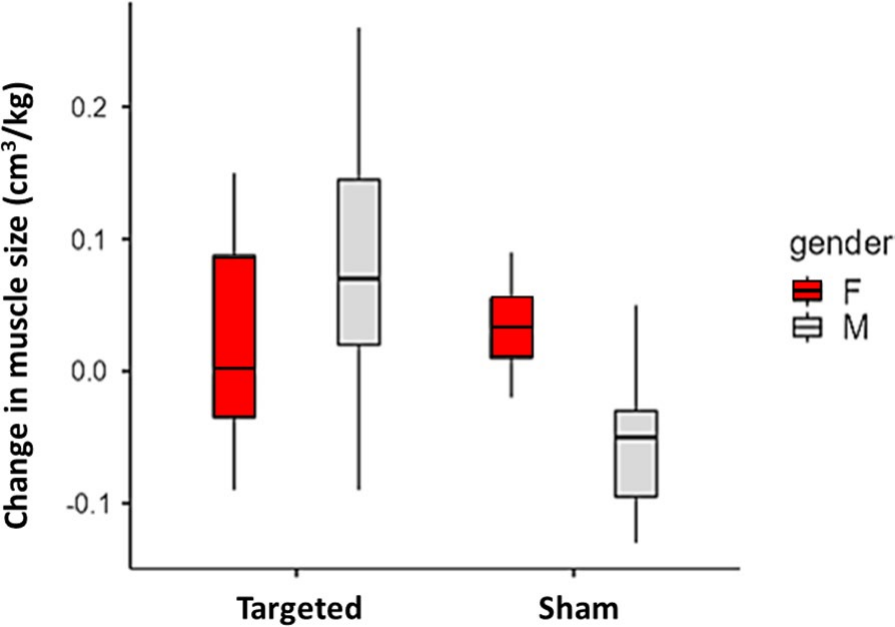
Normalised GMin muscle size differences (post-intervention – baseline, cm^3^/kg) for the affected limb comparing females (red) and males (grey) across the targeted and sham interventions. Note. Box represents interquartile range (IQR), median line, minimum and maximum values

For the affected limb, the increase in GMin volume following the targeted intervention was more pronounced for male participants in contrast to the sham intervention (sex x group effect: F_1,23_ = 5.32, *P* < 0.03; Fig. 4). Post-hoc analysis indicated an increase in GMin muscle volume for males that were allocated to the targeted gluteal intervention compared to males in the sham intervention with no difference between groups for female participants. No sex differences existed in affected limb for GMed, GMax and TFL muscle volumes in response to the interventions (sex main effect: F_1,23_ ≤ 0.96, *P* ≥ 0.34).

### Muscle fat index

**Fig. 5:**
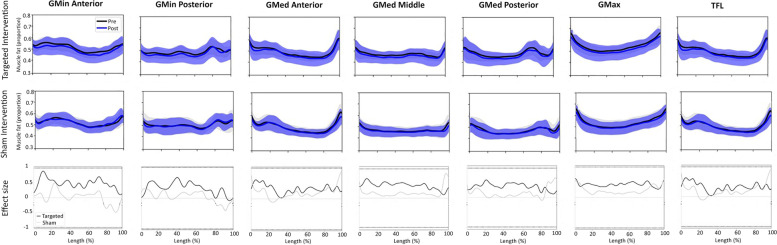
Group means and SD for proportion of fat along the length of GMin and GMed muscle segments and GMax and TFL muscles at baseline (black line) and post-intervention (blue line) (0% to 100% = proximal to distal) and effect size (ES) for differences in fat proportion following the targeted gluteal and sham interventions. Note. Solid black line represents ES for targeted gluteal intervention and light grey line for the sham intervention post-intervention along the length of the muscle

The pattern of change from baseline to post-intervention did not differ by intervention (time x group effect: all *P* ≥ 0.05) and no significant group or time effects existed for fatty infiltrate in all muscle segments in the affected limb following the targeted intervention (Fig. 5). Effect sizes for the difference between baseline and post-intervention observed for all muscles along the entire length of the muscles ranged between ES = 0.32 to 0.47 following the targeted intervention compared to ES = 0.02 to 0.23 for the sham intervention (Fig. 5).

### Isometric hip muscle strength

**Fig. 6:**
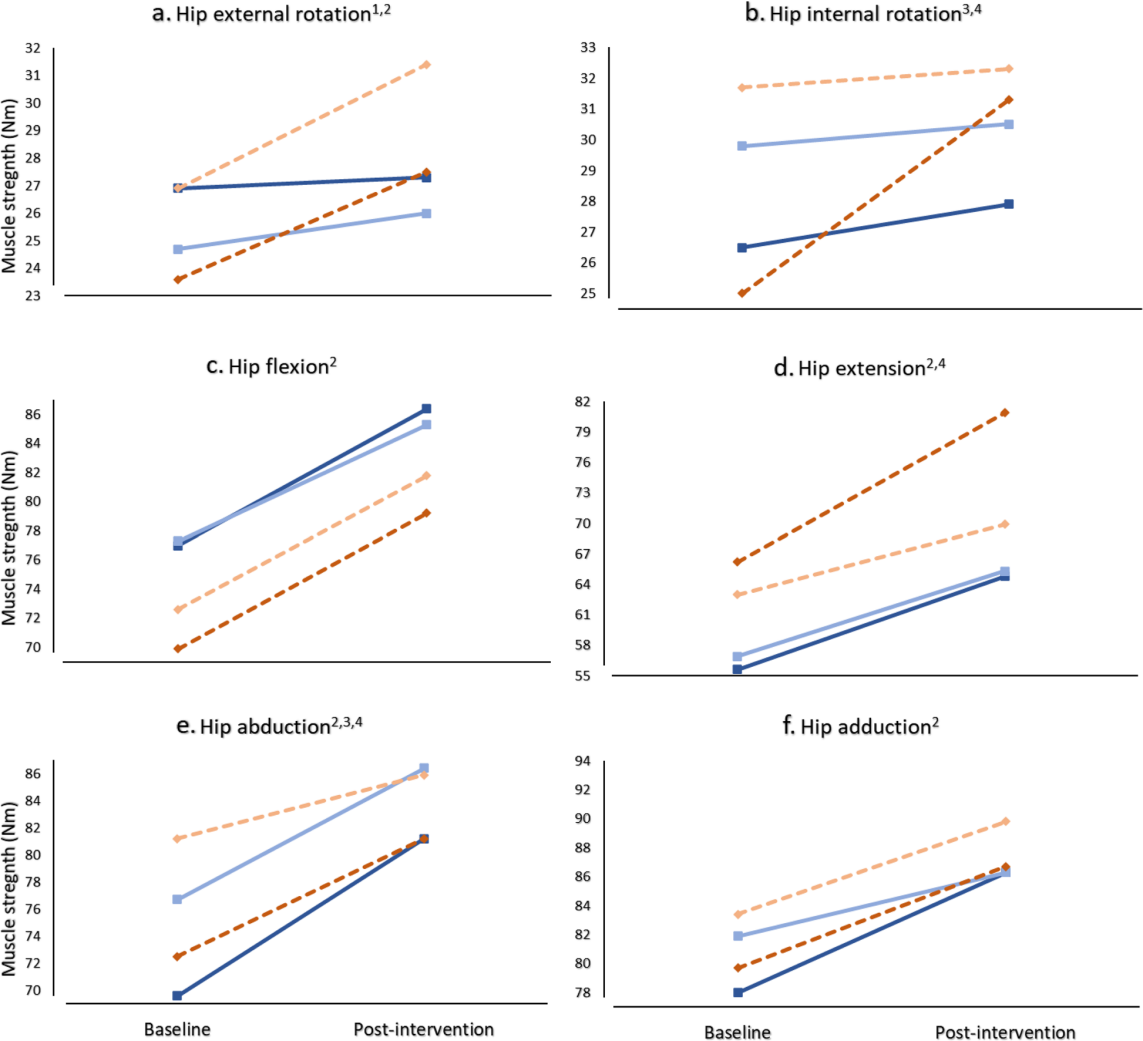
**a**-**f** Mean isometric hip muscle strength (Torque, Nm) for the targeted (solid blue lines) and sham interventions (dashed orange lines). Note. Affected and contralateral limbs indicated by darker and lighter lines, respectively (standard deviations in Additional file 2). ^1^Limb x group effect (*p* < 0.05); ^2^ Time main effect (*p* < 0.05); ^3^ Limb main effect (*p* < 0.05); ^4^ Data transformed to achieve normality

The pattern of changes in hip strength measurements from baseline to post-intervention were not different between groups (time x group effect: F_1,25_ ≤ 1.91, *P* ≥ 0.179; Fig. 6) with effect sizes that ranged between ES = 0.00 to 0.51 for all strength measures in the affected limb and ES = 0.02 to 0.43 in the contralateral limb (see Additional file 2). Isometric hip muscle strength increased from baseline to post-intervention in both groups (time main effect) for hip external rotation (F_1,25_ = 4.60, *P* = 0.042, pooled MD: 2.51 Nm, 95% CI: 0.10 to 4.94), flexion (F_1, 25_ = 7.19, *P* = 0.013, pooled MD: 8.97 Nm, 95% CI: 2.08 to 15.86), extension (F_1,25_ = 6.46, *P* = 0.018, pooled MD: 0.09 Nm, 95% CI: 0.02 to 0.17), abduction (F_1,25_ = 11.83, *P* = 0.002, pooled MD: 0.07 Nm, 95% CI: 0.03 to 0.11) and adduction (F_1,25_ = 5.94, *P* = 0.022, pooled MD: 6.54 Nm, 95% CI: 1.01 to 12.07). Lower strength was identified in the affected limb when compared to the contralateral limb at both baseline and post-intervention (limb main effect) for internal rotation (F_1,25_ = 11.59, *P* = 0.002, MD: -0.34 Nm, 95% CI: -0.54 to -0.13) and abduction (F_1,25_ = 7.14, *P* = 0.022, MD: -0.04 Nm, 95% CI: -0.07 to -0.01).

**Fig. 7:**
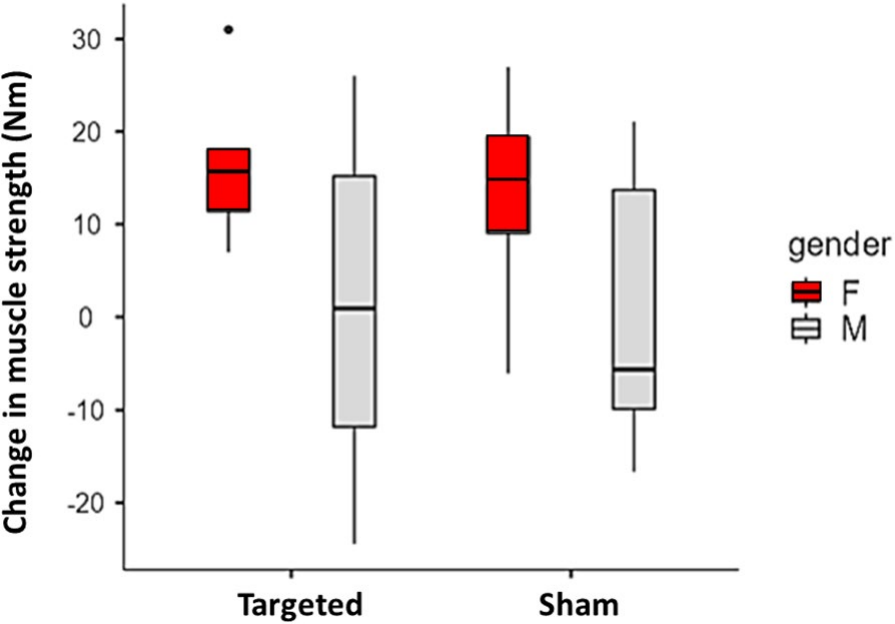
Isometric hip adduction strength differences (post-intervention – baseline, Torque, Nm) for the affected limb comparing females (red) and males (grey) following the targeted and sham interventions. Note. Box represents interquartile range (IQR), median line, minimum and maximum values with any outliers

No sex differences existed between interventions for hip strength in the affected limb (sex x group effect: F_1,23_ ≤ 3.34, *P* ≥ 0.08). There was an increase in hip adduction strength, demonstrating a larger difference irrespective of intervention, for females when compared to males (sex main effect: F_1,23_ = 6.27, *P* = 0.02; Fig. 7).

## Discussion

The targeted gluteal intervention resulted in a significant increase in GMin muscle volume for both the affected and contralateral limbs, with similar patterns observed for GMed, GMax and TFL. The increase observed in GMin muscle volume in response to the targeted gluteal intervention was greater in males compared to females. While there were no significant reductions in the proportion of fatty infiltrate following either intervention, the targeted intervention resulted in lower levels of fatty infiltrate with moderate effect sizes across all muscle segments (Fig. 5). Strength increases were observed in the affected and contralateral limbs in both groups for hip abduction, adduction, external rotation flexion and extension.

The GMin hypertrophy observed in the targeted group likely reflects the exercises undertaken during the targeted gluteal intervention. Exercises (e.g., split squat with hip internal rotation resistance) were designed to target known deficits in the gluteal muscles in hip OA, specifically GMin, which shows greater levels of atrophy and fatty infiltration earlier in hip OA [2, 10, 33]. Exercises that involve hip internal rotation generate higher levels of GMin muscle activity [34] and likely contribute to muscle hypertrophy.

The initial aim of the targeted intervention was to recruit GMin, and on average the highest level of the GMin strengthening phase was reached early in the intervention and remained a focus for the remaining eleven weeks (on average) of the intervention. In contrast only two-thirds of participants reached the highest level of the pelvic stability phase and, on average, this was commenced with only five weeks of the program remaining. This may not have allowed sufficient time for participants to achieve hypertrophy of the other gluteal muscles. This might explain the consistent, but non-significant, pattern of change in GMed, GMax and TFL muscle volumes with small to large effect sizes. A longer intervention to enable more participants to progress to the more intense stages of the targeted intervention might have resulted in significant increases in the volume of the remaining gluteal muscles.

Increases in hip muscle strength (i.e., hip external rotation, hip flexion, hip extension, hip abduction and hip adduction) for both the affected and contralateral limbs of participants across both intervention groups was not anticipated but is consistent with the notion that non-specific exercise interventions can improve hip muscle strength in people with hip OA [35–37]. The improvement observed in muscle strength across both limbs and groups in the current study at post-intervention could be attributed to potential neuromuscular adaptations, such as motor learning via increased efficiency in motor unit recruitment as a result of short term muscle activation during exercise [38, 39], which may not be linked to a change in muscle size. There are other possible explanations for bilateral increases in strength. Firstly, the high rate of bilateral hip OA across all participants and possible adoption of prescribed exercises in both the affected and contralateral limbs. Secondly, the sham intervention did include exercises, albeit not targeted at gluteal muscles and a standardised education component that included general benefits of exercise that may have prompted increased exercise participation.

This study identified some sex related differences for both muscle volume and strength outcomes and this has previously been reported in a hip OA population [35]. Sex can impact gait kinematics and kinetics in people with hip OA, for example gait in men involves reduced hip adduction and greater forward trunk lean when compared to women [40]. It is plausible that the increase in hip adduction strength noted in women when compared to men might relate to previously reported sex differences in coronal plane gait biomechanics [40]. It is also possible that sex differences account for the greater increase in GMin hypertrophy for males in the targeted group. However, these sex differences are observed in relatively small sample sizes in the current study.

Greater amounts of fatty infiltration in the gluteal muscles have previously been observed in hip pathology populations (i.e., hip OA and total hip arthroplasty) [41–43]. The similar pattern of reduced fatty infiltrate across all muscle segments following the targeted intervention is consistent with a recent study showing a decrease in gluteal muscle fat index in GMin and GMax in younger people with hip related groin pain following exercise programs [26]. The trend of reduced fatty infiltration, combined with increased GMin volume and improved hip muscle strength in the current study, is consistent with the notion that resistance based exercise training can reduce intramuscular fat [44] and improve overall muscle function (i.e., muscle strength) [45, 46].

This is the first study in people with hip OA to show that a targeted gluteal resistance exercise intervention can improve known gluteal muscle deficits, specifically in GMin, which have previously been demonstrated to occur in the early stages of this disease. However, the increase in GMin volume in the targeted group in the current study (approximately 4%) is similar to the volume increase reported following an exercise intervention in a previous study with a younger pathological population [26]. Similarly, the increase in hip abduction strength reported in the current study (approximately 12%) was consistent with previous exercise intervention studies [26, 35, 36]*.* A strength of this study was that all participants, researchers and outcome assessors were blinded to the intervention allocation until data analyses were completed. Additional confidence in the results is provided by the excellent inter-rater reliability (> 0.9) for all muscles traced in this study. The similar, but non-significant, patterns of muscle volume changes with some medium and large effect sizes in the remaining gluteal muscles suggest that a larger sample size might have resulted in more consistent findings for all muscles.

Failure to achieve statistically significant changes in fatty infiltration within the gluteal muscles might be partially explained by the limited sample size, as well as the early stage of disability and severity of disease recorded in participants of the current study. While the participants only had mild-to-moderate OA, there was evidence of relative atrophy of GMax in the affected limb, which is consistent with previous reports of muscle atrophy in hip OA [47]. However, improvements in strength and muscle volume were demonstrated despite dropout of one participant in the targeted group following five sessions in this intention-to-treat analysis. It is acknowledged that the results identified in the current study may not be generalisable to people with more severe hip OA. It is also possible that findings of this study may be subject to selection bias [48] by recruitment of participants more motivated to improve their muscle strength and physical fitness by volunteering to participate in a study that is known to contain an exercise program.

## Conclusion

A targeted gluteal intervention resulted in GMin muscle hypertrophy in both the affected and contralateral limbs and a pattern suggesting reduced fatty infiltrate in the affected limb of people with mild-to-moderate hip OA. Hip muscle strength increased following both interventions. Clinicians should consider exercises that target the gluteal muscles to reverse known atrophy in GMin and reductions in hip muscle strength. Future studies could investigate the relationships between gluteal muscle size and strength with clinical symptoms in people with mild-to-moderate hip OA.

## Supplementary Information

**Additional file 1. **Normalised muscle size (cm^3^/kg) for targeted and sham interventions across affected and contralateral limbs at baseline and post intervention, represented as mean ± SD.

**Additional file 2. **Muscle strength (Torque, Nm) for targeted and sham interventions across affected and contralateral limbs at baseline and post intervention, represented as mean ± SD.

## Data Availability

The datasets used and/or analysed during the current study are available from the corresponding author on reasonable request.

## Abbreviations

OA: Osteoarthritis
GMin: Gluteus minimus
GMed: Gluteus medius
GMax: Gluteus maximus
TFL: Tensor fasciae latae
GHOst: Gluteal exercise for Hip Osteoarthritis
MRI: Magnetic resonance imaging
OHS: Oxford hip score
