# Supplementary material for: Effects of a targeted resistance intervention compared to a sham intervention on gluteal muscle hypertrophy, fatty infiltration and strength in people with hip osteoarthritis: analysis of secondary outcomes from a randomised clinical trial
Source: BMC Musculoskelet Disord. 2022 Oct 29;23:944. doi: 10.1186/s12891-022-05907-4 (PMC9617418; doi:10.1186/s12891-022-05907-4)
Supplement: Supplementary file 1 — Additional file 1. Normalised muscle size (cm3/kg) for targeted and sham interventions across affected and contralateral limbs at baseline and post intervention, represented as mean ± SD. [file 12891_2022_5907_MOESM1_ESM.pdf]

Additional file 1. Normalised muscle size (cm^3/kg) for targeted and sham interventions across affected and contralateral limbs at baseline and post-intervention, represented as mean ± SD.

	Targeted (N = 13)				Sham (N = 14)					
	Affected limb		Contralateral limb		Affected limb		Contralateral limb			
	Baseline	Post-intervention	Baseline	Post-intervention	Baseline	Post-intervention	Baseline	Post-intervention	Between groups effect size for affected limb	Between groups effect size for contralateral limb
Gluteus Minimus (GMin)[1]	1.15 ± 0.18	1.21 ± 0.19	1.21 ± 0.19	1.25 ± 0.23	1.27 ± 0.32	1.26 ± 0.34	1.28 ± 0.30	1.25 ± 0.32	0.70	0.87
Gluteus Medius (GMed)	3.93 ± 0.58	3.98 ± 0.39	3.90 ± 0.59	3.96 ± 0.53	4.10 ± 0.66	3.94 ± 0.67	4.25 ± 0.73	4.15 ± 0.72	0.64	0.47
Gluteus Maximus (GMax)[2]	10.76 ± 2.25	10.91 ± 2.12	10.99 ± 2.29	11.15 ± 2.23	10.46 ± 1.32	10.36 ± 1.28	11.46 ± 1.76	11.33 ± 1.73	0.43	0.59
Tensor Fascia Lata (TFL)	0.88 ± 0.26	0.90 ± 0.25	0.82 ± 0.24	0.82 ± 0.22	0.81 ± 0.26	0.77 ± 0.23	0.82 ± 0.30	0.79 ± 0.29	0.94	0.40

[1] time x group effect ($P < 0.05$); [2] limb main effect ($P < 0.05$)
