# Supplementary material for: Effects of a targeted resistance intervention compared to a sham intervention on gluteal muscle hypertrophy, fatty infiltration and strength in people with hip osteoarthritis: analysis of secondary outcomes from a randomised clinical trial
Source: BMC Musculoskelet Disord. 2022 Oct 29;23:944. doi: 10.1186/s12891-022-05907-4 (PMC9617418; doi:10.1186/s12891-022-05907-4)
Supplement: Supplementary file 2 — Additional file 2. Muscle strength (Torque, Nm) for targeted and sham interventions across affected and contralateral limbs at baseline and post intervention, represented as mean ± SD. [file 12891_2022_5907_MOESM2_ESM.pdf]

Additional file 2. Muscle strength (Torque, Nm) for targeted and sham interventions across affected and contralateral limbs at baseline and post-intervention, represented as mean ± SD.

	Targeted (N = 13)				Sham (N = 14)					
	Affected limb		Contralateral limb		Affected limb		Contralateral limb			
	Baseline	Post-intervention	Baseline	Post-intervention	Baseline	Post-intervention	Baseline	Post-intervention	Between groups effect size for affected limb	Between groups effect size for contralateral limb
External Rotation[1, 2]	26.9 ± 7.8	27.3 ± 9.1	24.7 ± 6.9	26.0 ± 8.9	23.6 ± 9.8	27.5 ± 8.6	26.9 ± 11.5	31.4 ± 12.1	0.51	0.43
Flexion[2]	77.0 ± 30.7	86.4 ± 28.4	77.3 ± 28.0	85.3 ± 27.9	69.9 ± 33.5	79.2 ± 24.4	72.6 ± 35.0	81.8 ± 33.2	0.00	0.07
Internal Rotation[3, 4]	26.5 ± 10.5	27.9 ± 10.8	29.8 ± 9.4	30.5 ± 8.4	25.0 ± 11.0	31.3 ± 13.9	31.7 ± 14.7	32.3 ± 12.7	0.56	0.02
Extension[2, 4]	55.6 ± 30.6	64.8 ± 30.1	56.9 ± 27.9	65.3 ± 25.0	66.2 ± 38.0	80.9 ± 37.8	63.0 ± 30.2	69.9 ± 27.2	0.25	0.06
Abduction[2, 3, 4]	69.6 ± 27.5	81.2 ± 18.5	76.7 ± 32.5	86.4 ± 22.6	72.5 ± 39.2	81.2 ± 36.3	81.2 ± 37.2	85.9 ± 34.4	0.17	0.32
Adduction[2]	78.0 ± 31.8	86.3 ± 24.0	81.9 ± 33.7	86.3 ± 26.9	79.7 ± 43.6	86.7 ± 36.2	83.4 ± 38.6	89.8 ± 39.4	0.08	0.12

[1], limb x group effect ($p < 0.05$); [2], time main effect ($p < 0.05$); [3], limb main effect ($p < 0.05$); [4], Data transformed to achieve normality
